# Echovirus 30, Jiangsu Province, China

**DOI:** 10.3201/eid1104.040995

**Published:** 2005-04

**Authors:** Ya Nan Zhao, Qing Wu Jiang, Ren Jie Jiang, Liang Chen, David S. Perlin

**Affiliations:** * Fudan University, Shanghai, China;; † Yancheng Center for Disease Prevention and Control, Yancheng, China;; ‡ Public Health Research Institute, Newark, New Jersey, USA

**Keywords:** enterovirus, phylogenetic analysis, echovirus 30, meningitis, research

## Abstract

An outbreak of aseptic meningitis occurred in the northern area of Jiangsu Province in China from January to July in 2003. A total of 1,681 cases were involved in this outbreak, and 99% of patients were <15 years of age. To identify the etiologic agent, 66 cerebrospinal fluid specimens were tested by cell culture. Eighteen showed an enteroviruslike cytopathic effect on MRC-5 human fetal diploid lung cells. An enterovirus primer-mediated reverse transcriptase–polymerase chain reaction, a standard neutralization assay, and sequencing of the complete capsid-encoding (VP1) gene identified the 18 isolates (FDJS03) as echovirus 30. At least a 10% difference was seen in nucleotide sequences of VP1 between FDJS03 isolates and other global strains of echovirus 30. Phylogenetic analysis based on complete sequences of VP1 was performed to further characterize the FDJS03 isolates. This report is the first to identify a distinct lineage of echovirus 30 as a probable cause of this outbreak.

## Abstract 2

Global increase in outbreaks due to E30 indicates that detailed understanding of the transmission and evolution of enteroviruses is urgently needed.

Enteroviruses circulate worldwide and are the most commonly identified cause of aseptic meningitis, particularly in infants and young children; 30,000–50,000 persons per year are hospitalized with aseptic meningitis in the United States (1). During the past decade, numerous outbreaks of enteroviral meningitis were documented throughout the world (2–5). In China, infections with other enteroviruses are reported more frequently because wild-type polioviruses were eradicated by the expanded immunization program in 1992. Outbreaks associated with nonpolio enteroviruses have been sequentially reported in recent years (6–8). Specific serotypes of etiologic agents were not identified in outbreaks involving nonpolio enteroviruses in China, since serotyping usually had no influence on clinical management of a given patient with an enteroviral infection. However, identification of the serotype and the molecular characteristics of the prevailing virus can provide valuable epidemiologic information in outbreak investigations. The serotype-specific immune status of the population, territorial competition among serotypes, and transmission efficiency of the virus may also be important factors influencing epidemiologic behavior of human enteroviruses (HEV) (9,10). An understanding of circulating virus strains in local regions would be essential in effectively controlling enteroviral infections.

An unusual outbreak of aseptic meningitis in 1,681 patients occurred in the northern area of Jiangsu Province in China from January to July in 2003. In this report, we provide evidence for a distinct lineage of echovirus 30 as the etiologic agent of this outbreak.

## Materials and Methods

### Specimen Collection

Lumbar puncture on admission was used to obtain 204 cerebrospinal fluid (CSF) specimens from 204 hospitalized patients in prefectural hospitals in whom aseptic meningitis had been diagnosed. After culturing for bacterial growth, 66 CSF specimens were available for virus isolation on 3 cell lines. All CSF specimens (≈2 mL per sample) were sent to our laboratory in sterile containers at 4°C, separated into aliquots, and stored at –80°C for further study.

### Cell Culture and Virus Isolation

Human fetal diploid lung (MRC-5), buffalo green monkey kidney (Vero), and human epidermoid carcinoma (HEp2) cells were used in the study. Cells were grown in minimal essential medium (MEM) supplemented with 10% newborn calf serum, 50 U/mL of penicillin, and 50 µg/mL of streptomycin. Viruses were isolated from the original clinical specimens and propagated in cell culture by standard methods (11). Briefly, 200 µL of each CSF specimen was added in duplicate into 24-well plates covered with monolayers of each cell culture. Maintenance medium (MEM plus 2% newborn calf serum) was then added to each well. All cultures were incubated at 37°C in an atmosphere of 5% CO_2_ and observed daily for 7 days for an enteroviruslike cytopathic effect (CPE). In cultures exhibiting no CPE by the end of observation period, blind passage was performed for another 7 days. Passage was performed twice before the culture was reported as negative. Cultures showing an enteroviruslike CPE were passed once more for confirmation. The primary identification of positive isolates as enterovirus was done by a reverse transcriptase-polymerase chain reaction (RT-PCR) with 2 pairs of enterovirus general primers (12–14), which detect nearly all types of enterovirus (12,15). The reference enterovirus strain used in this study was coxsackievirus B1. Positive isolates were designated Echo30-FDJS03, which was abbreviated as FDJS03.

### Neutralization Assays with an Antibody Pool

Serotype identification was performed by neutralization with an antibody pool for enterovirus (Kunming Medical Biology Institute, Kunming, China) and 11 other type-specific monoclonal antibodies (Kunming Medical Biology Institute) not included in the antibody pool. Each positive strain was tested by a neutralization assay, according to standard procedures (16), and viral titers were simultaneously determined.

### Extraction of Viral RNA

Viral RNA was extracted from 200 µL of positive culture supernatants by using RNAex reagent (Huashun Co., Shanghai, China), according to the manufacturer's instructions, and diluted in 20 µL of diethylpyrocarbonate-treated sterile distilled water. The viral RNA solution was used immediately or stored at –80°C until analysis by RT-PCR. A standard reference virus strain and a normal cell culture supernatant were used as positive and negative controls, respectively.

### RT-PCR and Sequencing of DNA

A 2-step RT-PCR was used. Synthesis of cDNA was done in a 20-µL reaction volume containing 2 mmol/L deoxynucleotide triphosphates, 20 U of RNAsin (Promega, Madison, WI, USA), 10 pmol of random hexamer, 200 U of Moloney murine leukemia virus reverse transcriptase (Promega), 4 µL of 5× RT buffer, and 5 µL of extracted RNA. The mixture was incubated at 37°C for 60 min and inactivated at 95°C for 5 min.

The highly conserved 5´ untranslated region (UTR) was used to detect enterovirus by PCR amplification (17). Two sets of amplification primers (12–14), UG52 (sense, 5´-CAAGCACTTCTGTTTCCCCGG-3´, nucleotides [nt] 168–188, Bastianni) and primer 2 (sense, 5´-TCCTCCGGCCCCTGAATGCG-3´, nt 445–464, coxsackie B1) (12), and a common antisense primer (UC53 5´-TTGTCACCATAACCAGCCA-3´, nt 588–606, Bastianni) were used to produce PCR products of 440 (UG52/UC53) and 155 (primer2/UC53) bp, respectively. The PCR protocol consisted of denaturation at 94°C for 5 min; extension for 35 cycles at 94°C for 45 s, 52°C for 45 s, and 72°C for 45 s; and final extension at 72°C for 7 min.

The capsid-encoding VP3 and VP1 genes and the 2A gene of enterovirus were amplified with PCR primers 008 (sense, 5´-GCRTGCAATGAYTTCTCWGT-3´, nt 2411–2430, PV1-Mahoney) and 011 (antisense, 5´-GCICCIGAYTGITGICCRAA-3´, nt 3408–3389, PV1-Mahoney) (9). PCR amplification was done for 35 cycles at 94°C for 30 s, 51°C for 30 s, and 72°C for 30 s. The RT-PCR products were analyzed by electrophoresis on 2% agarose gels containing 0.5 µg/mL of ethidium bromide, and purified by using a gel extraction kit (Bioasia Co., Shanghai, China).

DNA sequencing was performed by using an automated DNA sequencer (ABI 3730, Applied Biosytems, Foster City, CA, USA). Each RT-PCR product was sequenced in both directions to resolve possible ambiguous nucleotides.

### Sequence Analysis

The serotypes of the viral isolates were determined by comparing their complete VP1 sequences with those available in the GenBank database. Nucleotide sequence homology was inferred by the identity scores obtained with the BLASTn program (National Center for Biotechnology Information, Bethesda, MD, USA). The pairwise sequence identities of the nucleotide and deduced amino acid sequences among FDJS03 isolates and other serotypes were calculated with the program Omiga 2.0 (Oxford Molecular Ltd., Madison, WI, USA). Four isolates randomly sampled from the 18 positive strains were used for phylogenetic analysis. Multiple-sequence alignments were done with ClustalX 1.83 (European Molecular Biology Laboratory, Heidelberg, Germany), and 2 methods (neighbor-joining and maximum parsimony) of phylogenetic analysis were used to provide a more reliable inference of phylogeny. A neighbor-joining tree was constructed with TREE-PUZZLE 5.0 (Deutsches Krebsforschungszentrum, Heidelberg, Germany) and the Dnapars program in software package PHYLIP 3.573c (University of Washington, Seattle, WA, USA) was used for maximum parsimony analysis. The statistical significance of phylogenetic trees constructed with Neighbor and Dnapars in PHYLIP 3.573c was estimated by bootstrap analysis with 100 pseudoreplicate data sets. The final consensus tree was produced with the Consense program in PHYLIP 3.573c. Treeview 1.6.6 (University of Glasgow, Glasgow, United Kingdom) was used to edit phylogenetic trees. The echovirus 30 sequences reported have been deposited in the GenBank database under accession nos. AY665606–AY665609.

## Results

An outbreak of aseptic meningitis in 1,681 patients occurred in the northern area of Jiangsu Province in China from January to July 2003. Most (99%) patients in this outbreak were children <15 years of age. Boys were nearly twice as likely as girls to have aseptic meningitis (ratio 1,145:536). Fever, headache, and vomiting were the most common clinical manifestations, and 204 CSF cultures were negative for bacterial growth. The epidemic was distributed in both urban and rural areas. The peak period was from March to June, 2003, when 1,565 cases occurred, which accounted for 93.1% of all patients.

To investigate the primary etiologic agent in this outbreak, CSF specimens were used for virus isolation and identification. Sixty-six CSF specimens from 66 aseptic meningitis patients were tested by cell culture, and 18 showed positive results (isolation rate 27.3%). An enteroviruslike CPE (data not shown) was observed in MRC-5 cells infected with these isolated strains, but not in Vero and HEp2 cells. Most positive isolates showed a CPE as early as 3–5 days postinoculation. The exception was 1 isolate that did not show any CPE until the third day of the second blind passage. For FDJS03 isolates, typical picornavirus particles (round, nonstructured virus particles ≈30 nm in diameter) were observed by negative staining and electron microscopy (data not shown). All 18 positive isolates were successfully amplified by RT-PCR with primers specific for enterovirus sequences to yield predicted products of 440 bp and 155 bp (data not shown). Specific serotype identification was performed with a standard microneutralization test, but it did not show neutralization of any strains with the antibody pool. This finding prompted serotype identification with additional monoclonal antibodies not present in the pool. The isolates were totally neutralized by monoclonal antibody to echovirus 30, and the other antibodies did not show neutralization. This finding suggests that all 18 positive strains were echovirus 30.

After the complete VP1-encoding genes of 4 randomly sampled isolates were sequenced, molecular identification of the isolates by comparison with corresponding sequences of HEV in GeneBank confirmed the results of neutralization assays. The percentage identity was calculated by pairwise comparisons of aligned nucleotide and amino acid sequences (Table). Among 4 FDJS03 isolates, 99% sequence identity was seen at both nucleotide and amino acid levels. HEV can be clustered into 4 species (HEV-A, HEV-B, HEV-C, and HEV-D) based on genetic relationships of the capsid-encoding region VP1 (1). The nucleotide and amino acid VP1 sequences of 4 FDJS03 isolates showed low identities with the HEV-A, HEV-C, and HEV-D enterovirus species. Identity scores for FDJS03 and other isolates with HEV-B were <70% and <80% at the nucleotide and amino acid sequence levels, respectively, except with echovirus 30 strains. According to proposed molecular typing criteria (1), the limits of intraserotypic divergence should be <25% nucleotide sequence differences or <12% amino acid sequence differences. FDJS03 isolates are clustered as echovirus 30 since they share high identities of both nucleotide (82%) and amino acid (92%) sequences with the prototype echovirus 30 Bastianni strain. This finding is in agreement with the results of the serotyping tests.

**Table:** Pairwise comparisons of nucleotide and amino acid sequences among FDJS03 isolates and other human enteroviruses (HEVs)*

Cluster†	Serotype	FDJS03_18		FDJS03_30		FDJS03_73		FDJS03_102	
		nt	aa	nt	aa	nt	aa	nt	aa
HEV-A	CA3	46	34	46	34	46	34	46	34
	CA4	44	32	44	31	44	31	44	32
	CA5	47	34	47	34	47	34	47	34
	CA6	47	33	47	33	47	33	47	33
	CA12	46	33	46	33	46	33	46	33
	CA14	42	29	41	29	42	29	42	29
HEV-B	CB2	60	61	60	61	60	61	60	61
	CB6	63	64	63	64	63	64	63	64
	E1	64	65	64	65	64	65	64	65
	E3	62	62	62	62	62	62	62	62
	E18	63	63	63	63	63	63	63	63
	E21	69	78	69	78	69	79	69	79
	E25	67	75	67	75	67	75	67	75
	**Echovirus 30**	**82**	**93**	**82**	**92**	**82**	**92**	**82**	**93**
	E32	61	62	61	62	61	62	61	62
HEV-C	CA1	49	43	49	43	49	43	49	43
	CA11	51	42	50	42	49	42	51	42
	CA15	50	42	50	42	50	42	50	42
	CA22	48	44	48	44	48	44	48	44
	CA24	49	40	49	40	49	40	49	40
	PV1	49	40	49	40	49	40	49	40
HEV-D	EV68	48	34	48	34	48	34	48	34

*nt, nucleotide identity score; aa, amino acid identity score; **boldface** used to highlight echovirus 30 serotype. †HEV-A was represented by CA3 (AF081294), CA4 (AF081295), CA5 (AF081296), CA6 (AF081297), CA12 (AF081302), and CA14 (AF081304); HEV-B was represented by CB2 (AF081312), CB6 (AF081313), E1 (AF081314), E3 (AF081316), E18 (AF081331), E21 (AF081334), E25 (AF081336), echovirus 30 (AF081340), and E32 (AF081345); HEV-C was represented by CA1 (AF081293), CA11 (AF081301), CA15 (AF081305), CA22 (AF081310), CA24 (AF081311), and PV1 (V01150); HEV-D was represented by EV68 (AF081348).

To investigate the genetic relationships between FDJS03 isolates and other echovirus 30 isolates from different regions and periods, a phylogenetic analysis was conducted on complete VP1 nucleotide sequences (Figure). The tree constructed by the neighbor-joining method is highly similar to that produced by maximum parsimony method (data not shown). According to the bootstrapping support values, echovirus 30 sequences segregate into 3 distinct groups (bootstrap value >80%), with some temporal and regional subclustering, similar to that in a previous study (9). FDJS03 isolates were monophyletic and closely related to each other, which suggests a common origin (bootstrap value 95). Sequences of FDJS03 isolates grouped in subgroup 3c together with those from Europe and America isolated in the 1970s and 1980s. Subgroup 3b was composed of viruses isolated between 1990 and 1998 in Europe and North America. In addition, subgroup 3a contained virus isolated from Japan in 1998 and those from an aseptic meningitis outbreak in Taiwan in 2001 that shared the same genotype with the other 2 subgroups, as observed in other studies (18,19). The prototype strain Bastianni and strains isolated before 1977 were distributed in 2 distinct genotypes (groups 1 and 2), which may have become extinct in recent years (9,18).

**Figure:**
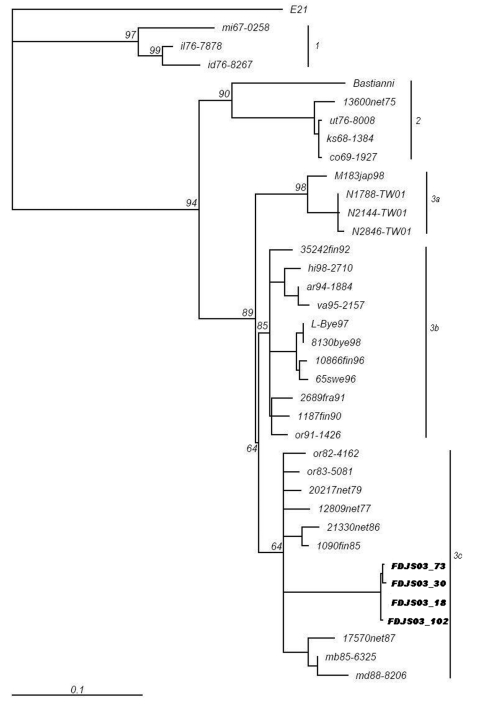
Phylogenetic tree based on complete VP1 sequences among FDJS03 isolates and other strains of echovirus 30 from different geographic and temporal origins. The neighbor-joining method was used to construct the tree. Numbers at the nodes represent the percentage of 100 bootstrap pseudoreplicates that contained clusters distal to the node. Prototype strain Farina of echovirus 21 (shown as E21) was included as the outgroup, but the tree is unrooted.

## Discussion

Echovirus 30 is one of the most frequently isolated enteroviral serotypes that causes aseptic meningitis. Numerous outbreaks of aseptic meningitis caused by echovirus 30 have been reported during the last decade in many countries (20–24). In the spring and summer of 2003, an aseptic meningitis outbreak occurred in the northern part of Jiangsu Province in China. Results of the present study show that a distinct lineage of echovirus 30 is likely responsible for this outbreak. In most cases, knowledge of a specific type of echovirus strain does not contribute to the management of patients. However, in large outbreaks, identifying enteroviral isolates from patient specimens should be of epidemiologic importance.

Enterovirus can be transmitted rapidly and may have caused worldwide infections (25). However, surveillance data for enterovirus are incomplete because of the voluntary nature of reporting and because only a small number of enteroviral isolates have been typed (9). The situation is much worse in many developing countries such as China. The number of reported outbreaks in China caused by nonpolio enteroviruses has increased in recent years, perhaps due to better surveillance (26), but little information is available for these viruses. The temporal dynamics and genetic diversity among strains of the echovirus 30 serotype have been reported in previous studies (1,9,27), but no echovirus isolates from China were included. The present study is the first to show that a distinct lineage of echovirus 30 caused meningitis in China. This finding is an important addition to the worldwide echovirus 30 information database.

In the present study, CSF specimens were obtained from only a small number of patients. These specimens were then tested by cell culture, largely because of logistic difficulties encountered since the peak period of this outbreak overlapped that of the epidemic of severe acute respiratory syndrome. We obtained a considerable number of echovirus 30 isolates, but no other serotypes of enterovirus. This finding strongly suggests that echovirus 30 was the etiologic agent in this outbreak.

The distinct lineage of echovirus 30 reported in this outbreak is not surprising, given that genomes of enteroviruses are known to evolve by ≈1%–2% per year (28–30), and interserotypic recombination contributes to evolution of these viruses (31–33). The FDJS03 isolates may represent possible recombinants between echovirus 30 and other serotypic enteroviruses. However, large antigenic variations in FDJS03 isolates were not observed, since they could be neutralized by standard antibodies to echovirus 30. A more detailed determination will require the complete sequencing of viral genome.

Phylogenetic analysis based on complete sequences of VP1 genes showed that FDJS03 isolates are closely related to each other, with an overall variation <1%. This finding indicates that this outbreak originated from a single genotype of echovirus 30. Unexpectedly, FDJS03 isolates are more closely related to echovirus 30 strains prevalent in Europe and North America than to those circulating in Japan and Taiwan. Group 3 echoviruses have been isolated in different areas of 3 continents, demonstrating the potential of this viral lineage to be transmitted over a large geographic region. Similarly, the temporal dynamics of echovirus 30 were seen both in Europe and North America, but until now no study reported the corresponding dynamics in Asia. Although we used 4 isolates from China and 3 from Japan and Taiwan in the phylogenetic analysis, this method is not sufficient to determine the overall genetic diversity and molecular dynamics of echovirus 30 in Asia. Nevertheless, pairwise comparison of nucleotide sequences and phylogenetic tree analysis demonstrated that the VP1 sequences of FDJS03 isolates exist in a subcluster composed of sequences from viruses isolated in North America in the early 1980s. However, these affinities seem less closely related between FDJS03 isolates and those circulating during the 1990s. Unfortunately, insufficient data are available to make definitive conclusions about defining characteristics of echovirus 30 in China and elsewhere.

In conclusion, we have characterized a distinct lineage of echovirus 30 strains isolated from CSF specimens of aseptic meningitis patients, which appears to be responsible for an outbreak in the northern area of Jiangsu Province in China in 2003. The VP1 sequences from these isolates are most similar to those of echovirus 30 strains that were common in North America in the early 1980s. This report is the first of the molecular characteristics of echovirus 30 strains circulating in mainland China and their respective phylogenetic relationships. However, we could not provide a comprehensive description of the genetic diversity and dynamics of echovirus 30 in China or Asia. Thus, establishing an enterovirus molecular surveillance system is needed in China to provide a better understanding of virus transmission and evolution.
